# The long-term revision risk of RSA-tested and non-RSA-tested TKA implants in The Netherlands: a Dutch arthroplasty register study

**DOI:** 10.2340/17453674.2026.45293

**Published:** 2026-02-16

**Authors:** Thies J N VAN DER LELIJ, Bart G PIJLS, Bart L KAPTEIN, Liza N VAN STEENBERGEN, Rob G H H NELISSEN, Perla J MARANG-VAN DE MHEEN

**Affiliations:** 1 Department of Orthopaedics, Leiden University Medical Center, Leiden; 2 Dutch Arthroplasty Register (Landelijke Registratie Orthopedische Interventies), ’s-Hertogenbosch; 3 Safety & Security Science and Centre for Safety in Healthcare, Delf University of Technology, Delft, The Netherlands

## Abstract

**Background and purpose:**

Radiostereometric analysis (RSA) of total knee arthroplasty (TKA) is used as an early safeguard during the phased evidence-based introduction of new implants. The goal of our study was to compare the long-term revision risk between RSA-tested implants and non-RSA-tested implants in the Netherlands using patient-level data.

**Methods:**

All primary TKAs between 2007 and 2016 from the Dutch Arthroplasty Register were included, and procedures with an RSA-tested implant were identified. Both all-cause major revision risk and revision risk because of loosening were calculated at 5 and 10 years postoperatively using Kaplan–Meier analyses. Sensitivity analyses were performed with stricter definitions of implant characteristics to classify procedures as RSA-tested, to avoid camouflage of different subdesigns within the same brand implant portfolio.

**Results:**

83,638 RSA-tested and 104,105 non-RSA-tested TKAs were included. Cumulative all-cause major revision percentages for the RSA-tested group at 5 and 10 years were 2.2% (95% confidence interval [CI] 2.1–2.3) and 3.6% (CI 3.4–3.7), respectively, compared with 2.5% (CI 2.4–2.6) and 3.3% (CI 3.2–3.4) for the non-RSA-tested group. RSA-tested TKAs showed higher 10-year revision risks because of loosening than non-RSA-tested procedures (1.8%, CI 1.7–1.9 vs 1.4%, CI 1.3–1.4, respectively). Comparable results were found after stratification by various patient characteristics and with stricter classification approaches.

**Conclusion:**

Regarding all-cause revision risk, RSA-tested TKAs had a slightly lower risk at 5 years. However, at 10 years the RSA-tested TKAs had a higher all-cause revision risk and higher revision risk because of loosening compared with the non-RSA-tested TKAs.

Radiostereometric analysis (RSA) is a valuable tool in the phased evidence-based introduction of arthroplasty implants [1-3]. Early implant migration results, obtained with RSA, can warn clinicians about implants that have an increased long-term risk of revision due to loosening. However, only a limited number of new implants have been studied with RSA. If TKAs with an RSA-tested implant show a lower long-term revision risk compared with TKAs using a non-RSA-tested implant, RSA testing of all implants introduced to the market could be advocated.

Hasan et al. recently reported that “RSA-tested” TKAs on average have a lower 10-year revision risk than “non-RSA-tested” TKAs [4]. They used the aggregate-level mean all-cause revision risk of TKAs from annual reports of national arthroplasty registries. Therefore, they could not assess whether the patient populations receiving RSA-tested and non-RSA-tested implants were comparable. Moreover, early migration as measured with RSA is used as a proxy for the risk of revision due to loosening, not for all-cause revision. Furthermore, Hasan et al. [4] did not take into account the differences between subtypes within the same implant brand portfolio [5,6]. We know that good results from a (sub)design may camouflage the actual clinical performance of another (sub)design within the same brand implant portfolio that performs worse [5]. This suggests that more detailed patient-level analyses are warranted.

The aim of our study was to compare both the long-term all-cause major revision risk and revision risk due to loosening between RSA-tested implants and non-RSA-tested implants in the Netherlands using patient-level data.

## Methods

### Study design

Patient-level data for our observational study were obtained from the Dutch Arthroplasty Register (LROI), to avoid spurious associations due to the ecological fallacy when using aggregate-level data (i.e., the erroneous inference about individuals based on findings for the group to which these individuals belong) [7]. This nationwide population-based register started in 2007 and achieved 100% coverage of all Dutch hospitals in 2012, and 96% completeness for primary TKAs and 90% of revision arthroplasty for knees in 2012 [8]. The LROI database contains patient, procedure, and implant characteristics. For each component, specific implant characteristics can be identified by its product number [9]. In the LROI, the product number of all prosthetic components and cement (if used) are registered. This allows the opportunity to extend the information concerning specific prosthesis, e.g., the specific bone-surface coating of the implant. To receive informed consent of patients, the LROI uses an opt-out system. The study is reported in accordance with the Strengthening the Reporting of Observational Studies in Epidemiology (STROBE) guidelines [10].

### Patient selection and classification

All primary TKAs performed between 2007 and 2016 registered in the LROI were included. To identify whether an implant was tested with RSA, the results of a previous meta-analysis on tibial baseplate migration were used [3]. In short, Pijls et al. [2,3] conducted a literature search to identify all clinical RSA studies on tibial component migration in TKA that were published up to 2016. The results of this search were used to classify procedures as “RSA-tested” (i.e., when an implant was used in a clinical RSA study) or “non-RSA-tested” (i.e., when an implant was not tested with RSA). Procedures up to 2016 were included in this study, which ensured that patients had at least 5 years of follow-up. The same database of RSA-tested implants was used by Hasan et al. [4].

The classification of TKAs was performed at 3 levels of specificity. For the primary analyses, implants were coded at the level of implant (brand) and fixation method (cemented/uncemented) (level 1). If a brand/fixation combination was described in an RSA study, the procedure in the LROI was coded as “RSA-tested”. Otherwise, the procedure was coded as “non-RSA-tested”. This level of coding was chosen for the primary analyses to align with previous research, but also because this level of information is mostly reported in RSA studies and revision risks in annual arthroplasty registry reports are reported on this level [1,4]. Moreover, we explored possible variation in revision risks between implants within the RSA-tested and non-RSA-tested group.

Furthermore, 2 additional, stricter definitions were used to classify implants as RSA-tested (levels 2 and 3). Besides the brand/fixation method, level 2 also included insert type (fixed/mobile bearing, cruciate retaining [CR]/posterior stabilized [PS]). To be classified as RSA-tested at level 3, implants had to match exactly with an RSA study at the subtype level within the implant brand portfolio, fixation method, fixation method specified (e.g., implant coating or type of cement), and insert type. If the implant that was used during TKA did not exactly match the implant described in an RSA study, based on the level criteria, the procedure was coded as non-RSA-tested at that level (see Table 2).

**Table 2:** Number of procedures classified as “RSA-tested” and “non-RSA-tested” based on 3 different levels of matching

Level of implant classification	RSA tested	non-RSA tested
Level 1 (brand/fixation)	83,638	104,105
Level 2 (brand/fixation/insert)	68,634	119,109
Level 3 (brand/subtype/fixation/fixation specified/insert)	37,788	149,955

We only classified TKAs as RSA-tested after publication of an RSA study in which the implant was studied. Only after implant migration results have been published is it possible for clinicians to estimate the risk of late revision due to loosening. Subsequently, clinicians may decide to continue or discontinue using a specific implant (based on its expected risk of late revision) resulting in a selection of best performing implants and thereby lower revision risks for RSA-tested implants. When no RSA migration results of an implant are available at the time of surgery, it is not possible to estimate the risk of future long-term loosening. In this way, some TKAs may be classified as non-RSA-tested if the surgery was performed before publication of the RSA study and other TKAs using the exact same implant as RSA-tested if the surgery was performed after publication of the RSA study.

### Statistics

All-cause major revision risks (i.e., revision of the femoral and/or tibial component) were calculated at 5 and 10 years for the RSA-tested and non-RSA-tested group (level 1) using Kaplan–Meier analyses. Minor revisions (i.e., change of polyethylene insert, insertion/change of patellar component, and DAIR without implant change) were not used in our analyses. Survival time was calculated as the time from primary TKA surgery to the first major revision, death of the patient, or end of follow-up (January 1, 2022). After visual inspection of the Kaplan–Meier plots and testing of scaled Schoenfeld residuals, the proportional hazards assumption was shown to be violated. Therefore, a time-stratified Cox model was fitted by splitting the follow-up period into 3 intervals (0–2, 2–7, and 7–10 years) and including an interaction term between group (RSA-tested vs non-RSA-tested) and time interval. This allowed estimation of hazard ratios (HR) specific to each clinically meaningful interval (early, mid-term, and long-term). To evaluate and control for possible confounders, and take these into account, we compared the all-cause major revision risks for both groups stratified by age group, sex, diagnosis, and American Society of Anesthesiologists (ASA) classification. The time-stratified Cox model was adjusted for these patient characteristics as well. We did not stratify by body mass index (BMI), Charnley score, or smoking status as these have only been registered in the LROI since 2014 (see percentages of missing data in Table 1). Revision risks of individual brand/fixation combinations included in the RSA-tested and non-RSA-tested groups were calculated to assess possible heterogeneity within the groups. In addition to all-cause major revision, the (major) revision risk because of loosening at 5 and 10 years was calculated for the RSA-tested and non-RSA-tested group. Finally, we performed sensitivity analyses with the stricter matching definitions (levels 2 and 3). Means were reported with 95% confidence intervals (CIs), as the latter allows a more easy and direct evaluation of clinical significance compared with the P value [11]. Analyses were performed using SPSS (version 29.0; IBM Corp, Armonk, NY, USA) and R software (version 4.2.1; R Foundation for Statistical Computing, Vienna, Austria).

**Table 1:** Baseline demographic characteristics. Values are count (%) or as specified

Factor	RSA-tested (n = 83,638)	non-RSA-tested (n = 104,105)
Age, mean (SD)	68.8 (9.2)	68.2 (9.6)
Available	83,521 (99.9)	104,001 (99.9)
BMI, mean (SD)	29.7 (5.0)	29.8 (5.1)
Available	40,814 (49)	38.368 (37)
Sex		
Male	28,230 (34)	35,579 (34)
Female	55,270 (66)	68,236 (66)
Missing	138 (0.2)	290 (0.3)
Diagnosis		
Osteoarthritis	79,806 (95)	98,366 (95)
Rheumatoid arthritis	1,253 (1.5)	1,721 (1.7)
Inflammatory arthritis	45 (0.1)	48 (0.0)
Late post-traumatic	1,069 (1.3)	1,621 (1.6)
Osteonecrosis	380 (0.5)	393 (0.4)
Tumor	7 (0.0)	70 (0.1)
Other	167 (0.2)	462 (0.4)
Missing	911 (1.1)	1,424 (1.4)
ASA class		
I	14,424 (17)	19,585 (19)
II	54,929 (66)	64,969 (62)
III–IV	11,061 (13)	13,833 (13)
Missing	3,224 (3.9)	5,718 (5.5)
Charnley score		
A	17,691 (21)	17,518 (17)
B1	14,045 (17)	12,600 (12)
B2	8,099 (9.7)	7,173 (6.9)
C	1,247 (1.5)	1,126 (1.1)
N/A (no osteoarthritis)	1,724 (2.1)	3,139 (3.0)
Missing	40,832 (49)	62,549 (60)
Smoking		
Yes	3,780 (4.5)	3,447 (3.3)
No	35,461 (42)	30,744 (30)
Missing	44,397 (53)	69,914 (67)
Previous surgery		
Yes	25,721 (31)	31,282 (30)
No	53,967 (65)	63,909 (61)
Missing	3,950 (4.7)	8,914 (8.6)

ASA = American Society of Anesthesiologists Physical Status classification, BMI = body mass index, N/A = not applicable.

### Data sharing plan, funding, use of AI, and disclosures

This investigator-initiated study was supported by a grant from Stryker. The sponsor did not take part in the design, conduct, analyses, or interpretations in the present study. AI tools were not used. Authors report no conflict of interests.Complete disclosure of interest forms according to ICMJE are available on the article page, doi: 10.2340/17453674.2026.45293

## Results

**Figure 1:**
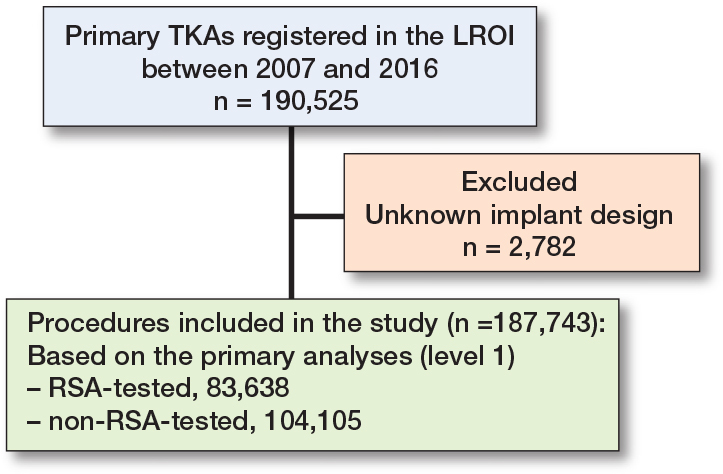
Patient flowchart.

190,525 primary TKAs between 2007 and 2016 were registered in the LROI. Procedures with an unknown implant design (n = 2,782) were excluded, leaving 187,743 procedures included in this study. Based on the classification for the primary analyses (level 1), 83,638 RSA-tested and 104,105 non-RSA-tested TKAs were included (Figure 1). Patient demographics were comparable between the groups (Table 1). As expected, most missing data were found for the variables BMI (RSA-tested 51%; non-RSA-tested 63%), Charnley score (RSA-tested 49%; non-RSA-tested 60%), and smoking (RSA-tested 53%; non-RSA-tested 67%), because they were not registered before 2014.

### All-cause major revision

**Figure 2:**
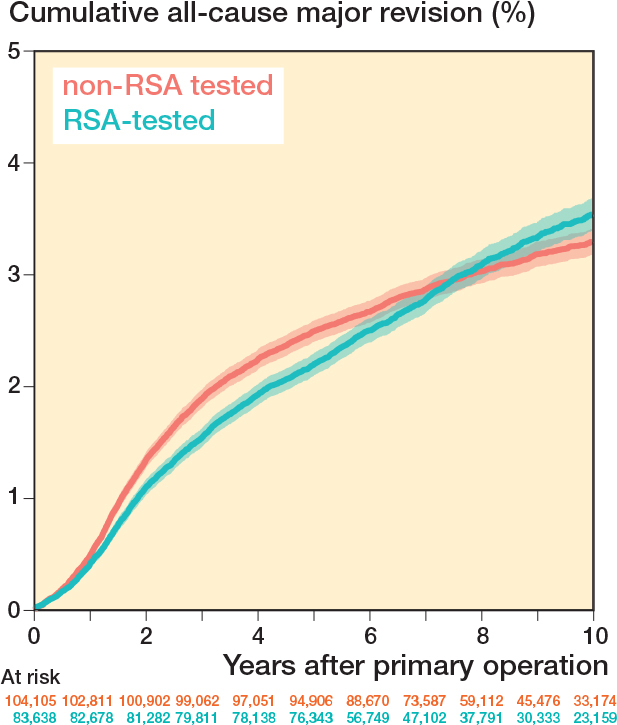
Cumulative all-cause major revision risk (Kaplan–Meier) through 10 years of follow-up for RSA-tested and non-RSA-tested implants.

**Figure 3:**
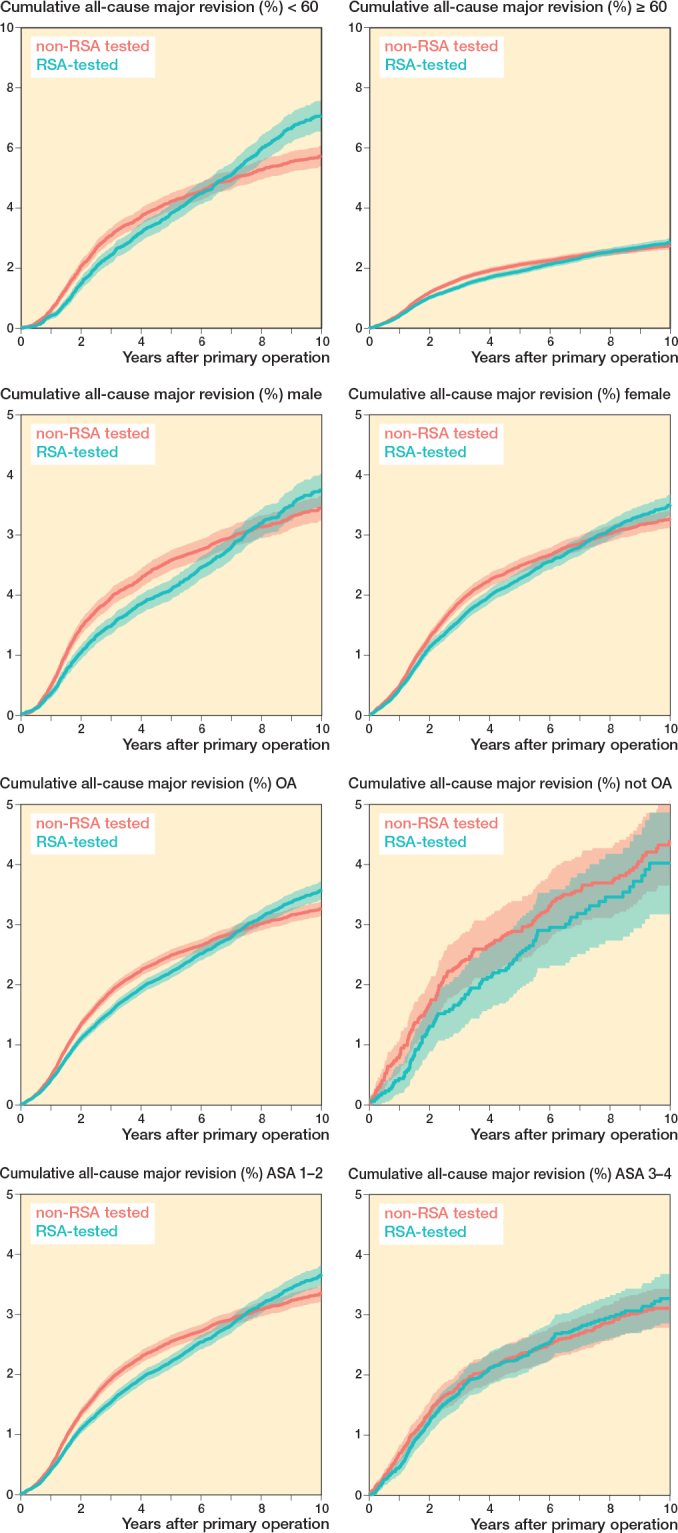
Cumulative all-cause major revision risk (Kaplan–Meier) plots through 10 years of follow-up for RSA-tested and non-RSA-tested procedures stratified by (A) age, (B) sex, (C) diagnosis, and (D) American Society of Anesthesiologists (ASA) classification.

**Figure 4:**
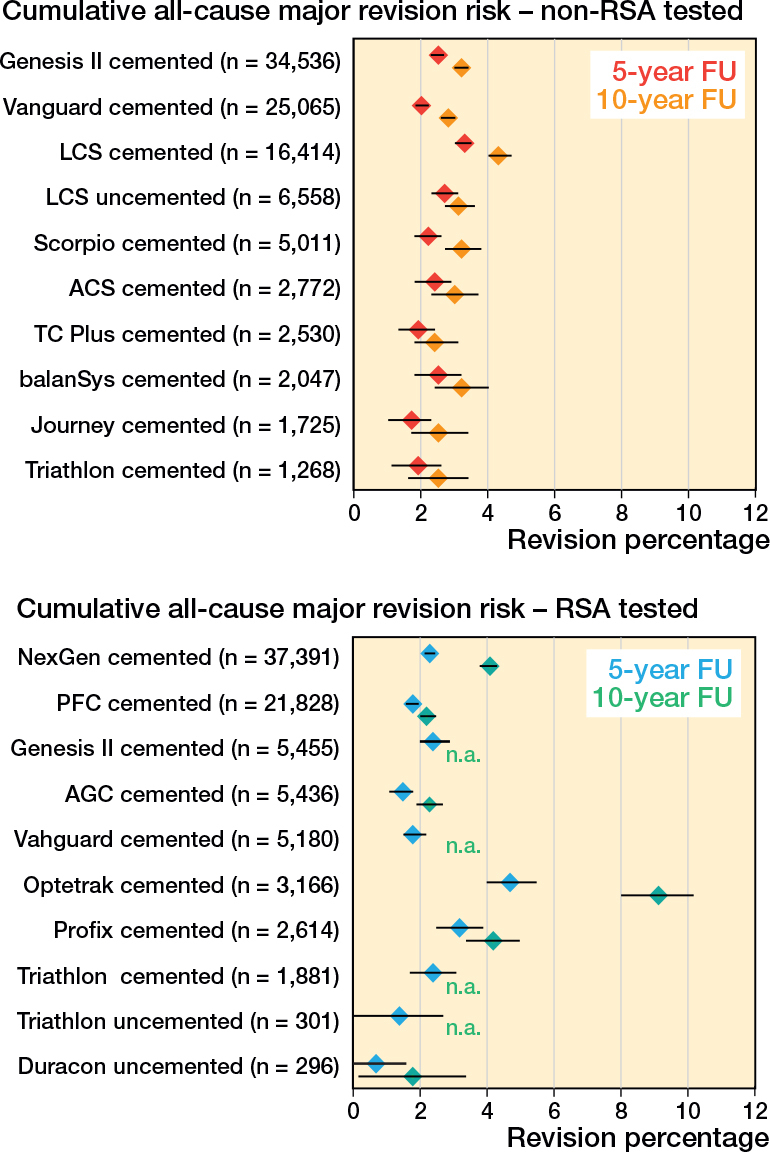
Forest plots showing the cumulative all-cause major revision risk (with 95% confidence intervals) of individual implant/fixation combinations within the RSA-tested and non-RSA-tested TKA group at 5 and 10 years postoperatively. For both groups, the 10 implant/fixation combinations with the greatest number of procedures is shown. Note: n.a. if < 50 cases were at risk.

Cumulative all-cause major revision risks for the RSA-tested group at 5 and 10 years were 2.2% (CI 2.1–2.3) and 3.6% (CI 3.4–3.7), respectively, compared with 2.5% (CI 2.4–2.6) and 3.3% (CI 3.2–3.4) for non-RSA-tested procedures (Figure 2). Revision risks in patients < 60 years were greater compared with patients > 60 years in both the RSA-tested and non-RSA-tested group (Figure 3). Still, after stratification by age, sex, diagnosis, and ASA classification, TKAs with an RSA-tested implant did not have lower long-term all-cause major revision risks. Within the RSA-tested group, revision risks at 10 years ranged from 1.8% (CI 0.2–3.4) (Duracon uncemented) to 9.1% (CI 8.0–10.2) (Optetrak cemented) with less variation in the non-RSA-tested group (ranging from 2.4%, CI 1.8–3.1 to 4.3%, CI 4.0–4.7) (Figure 4). The time-stratified Cox model adjusted for patient characteristics showed that the hazard ratio (HR) in the first 2 years postoperatively was 0.86 (CI 0.79–0.94, P < 0.001), indicating a lower risk of revision for the RSA-tested group. However, between 2 and 7 years the HR was 1.14 (CI 1.05–1.23, P = 0.001) and between 7 and 10 years the HR was 2.02 (CI 1.69–2.41, P < 0.001), indicating a higher risk of revision for the RSA-tested group.

### Major revision due to loosening

**Figure 5:**
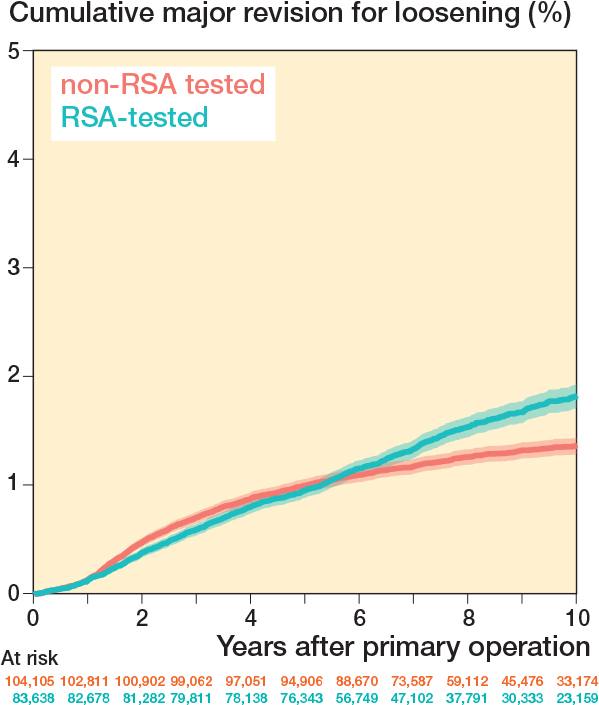
Cumulative major revision risk because of loosening (Kaplan–Meier) through 10 years of follow-up for RSA-tested and non-RSA-tested implants.

Cumulative major revision risks because of loosening for the RSA-tested group at 5 and 10 years were 1.0% (CI 0.9–1.0) and 1.8% (CI 1.7–1.9), respectively, compared with 1.0% (CI 0.9–1.1) and 1.4% (CI 1.3–1.4) for non-RSA-tested group (Figure 5).

### Stricter classification of RSA-tested procedures

When using stricter definitions to match implants with clinical RSA studies, fewer TKAs were classified as RSA-tested (Table 2). Of the 187,734 primary TKAs included in the study, 68,634 were classified as RSA-tested at level 2. Using the latter classification, the cumulative all-cause major revision risk for the RSA-tested group at 10 years was 3.7% (CI 3.5–3.9) compared with 3.3% (CI 3.2–3.4) for the non-RSA-tested group. For major revision due to loosening, the 10-year revision risk was 2.0% (CI 1.8–2.1), and 1.3% (CI 1.3–1.4) for the RSA-tested and non-RSA-tested group, respectively.

At the strictest level of classifying the implants (level 3), 37,788 procedures were classified as RSA-tested. With this classification, the cumulative all-cause major revision risk for the RSA-tested group at 10 years was 4.0% (CI 3.7–4.2) compared with 3.3% (CI 3.2–3.4) for non-RSA-tested group. The mean 10-year revision risk due to loosening was 2.1% (CI 1.9– 2.3) for the RSA-tested and 1.4% (CI 1.4–1.5) for non-RSA-tested group.

## Discussion

The goal of our study was to compare the long-term revision risk between RSA-tested implants and non-RSA-tested implants in the Netherlands using patient-level data. At 10 years’ follow-up, the cumulative all-cause major revision risk in the RSA-tested group was higher than in the non-RSA-tested group. At 10 years’ follow-up, the risk of revision because of loosening was also higher in the RSA-tested group than in the non-RSA-tested group. These findings remained consistent after stratification by demographic characteristics and using stricter definitions for the classification of TKAs as RSA-tested.

Interestingly, within the RSA-tested group, revision risks between individual brand/fixation combinations varied considerably. The highest revision risk was observed in TKAs using the Optetrak cemented implant (10-year cumulative all-cause major revision risk 9.1%, CI 8.0–10.2). Because an RSA study of the Optetrak implant was published in 2004, all procedures in the LROI with this implant were classified as RSA-tested [12]. However, this RSA study does not explicitly conclude that there is an increased risk of long-term revision for this specific implant based on the early migration data. This may be due to the fact that mean implant migration thresholds predictive of late loosening to enable classification of acceptable and unacceptable migration were only proposed in 2012 [2,12]. Based on these thresholds, both the cemented Optetrak CR and PS implants would be considered to be “at risk” of revision risks higher than 5% at 10 years. Another possible explanation for high revision risks despite RSA testing may be that (seemingly) small changes to an implant design, its manufacturing process, or packaging method have been made, which may influence the clinical performance of an implant and increase the revision risk. For the Optetrak knee system, the polyethylene (PE) inserts manufactured since 2004 have been recalled because they were packaged in bags that did not contain a secondary barrier layer with ethylene vinyl alcohol to augment oxygen resistance. The use of these non-conforming bags increased oxygen diffusion to the ultra-high molecular weight polyethylene (UHMWPE) inserts, which can severely damage their mechanical properties and lead to accelerated wear debris and subsequently bone loss and loosening of the TKA implant [13]. Therefore, one could argue that the Optetrak implants included in the RSA study are essentially different implants compared with the Optetrak implants used later and registered in the LROI. It has been shown previously that (accidental) changes in the manufacturing process of orthopedic implants may significantly affect their clinical performance and that the type of PE insert material may influence the revision risk after TKA [14,15]. On the other hand, other designs may also have changed after the RSA study was published, influencing their performance, which could be explored in future studies.

RSA assesses implant migration, which has been associated with long-term revision risk due to loosening [2]. However, early implant migration as measured with RSA has not been associated with revision for other reasons, such as infection or implant/liner wear. Furthermore, besides revision rates, patient-reported outcome measures (PROMs) are increasingly employed in orthopedic practice to assess clinical outcome after TKA. We have previously shown that TKA migration, as measured with RSA, is not associated with postoperative changes in PROMs or clinical scores [16]. Therefore, the performance and safety of novel TKA implant designs cannot be assessed on RSA alone.

We found that the mean revision risk at 10 years’ follow-up of non-RSA-tested TKAs in the Netherlands was very low. This is consistent with the results from Hasan et al. [4], who showed that the revision risk of non-RSA-tested TKAs in the Netherlands was lower compared with non-RSA-tested TKAs in other countries [4]. This may be due to the fact that the Netherlands has a long tradition of ensuring the quality of orthopedic implants used, first through a national framework established by the Dutch Orthopaedic Society (NOV) that critically evaluated clinical data and performance of implants during annual meetings, later followed by the use of the Orthopaedic Data Evaluation Panel (ODEP) ratings [17]. In other countries, with less market regulation and no systems in place to ensure the quality of new implants, long-term revision risks of non-RSA-tested implants may be higher and RSA testing may have a larger impact to ensure good quality implants so that procedures with an RSA-tested implant would show lower long term-revision risks consistent with our hypothesis.

We have shown that the 10-year revision risks because of loosening of both RSA-tested and non-RSA-tested implants are low. Therefore, one may argue that fixation is not the big problem anymore in contemporary TKA. While mean revision percentages are small, the absolute number of patients requiring revision surgery because of loosening remains large; given the large amount of primary arthroplasty procedures performed each year.

### Strengths

A strength of our study is that it is based on a large real-world population-based cohort (registry) with very high completeness. Utilizing patient-level data obtained from the LROI allowed us to gauge the impact of baseline characteristics by making stratified comparisons between groups. Furthermore, it was possible to take into account the time of surgery and year of publication of RSA studies when classifying TKAs as RSA-tested or non-RSA-tested, to mimic the mechanism through which RSA-testing might have an effect. Besides addressing the possible camouflage effect by using stricter definitions for the classification of implants, we also looked specifically at the revision risks due to loosening rather than all-cause revision, as early migration measured with RSA is associated with revision due to loosening.

### Limitations

Our study was observational and therefore subject to confounding, such as confounding by indication and unmeasured patient- and surgeon-related factors. However, we found that the RSA-tested and non-RSA-tested groups were comparable regarding most of the observed patient demographics. Also, one could argue that additional time restrictions should be applied for the classification of implants as RSA-tested, because small changes to an implant design, after publication of an RSA study, may affect its clinical performance. For that matter, only procedures performed within < 10 years after publication of an RSA study on the specific implant that was used may be assumed to be the same implant and therefore classified as RSA-tested. However, such a time restriction would be arbitrary, as it is often unknown if and when such changes are made to an existing implant that is already on the market. We used data from a previous meta-analysis on clinical RSA studies, in accordance with the previous study by Hasan et al. [4]. However, this database includes only all published studies on tibial component migration in TKA and might miss potential clinical RSA studies that reported only femoral component migration. Finally, we compared the risk of revision because of loosening between the RSA-tested and non-RSA-tested group and intentionally did not use the terminology “aseptic” loosening. The reason for revision in the LROI is classified by the surgeon at the time of (revision) surgery and multiple reasons can be given (e.g., both infection and loosening). There is no feedback mechanism to capture cases that were initially not classified as infection, but with intraoperative cultures later showing that a (low-virulent) microbe may have led to the revision. On the other hand, a surgeon may intraoperatively suspect an infection as the reason for the revision, but with intraoperative cultures remaining negative.

### Conclusion

TKAs using an RSA-tested implant did not show lower revision risks at 10 years’ follow-up than TKAs using a non-RSA-tested implant in the Netherlands.

*In perspective*, although we found no beneficial association of RSA testing and long-term revision risk, we caution against interpreting the results of our study as suggesting that RSA studies are unnecessary or should no longer be conducted as results may be different in other countries.

## Notes

TL: Conceptualization, methodology, formal analysis, data curation, writing – original draft. BP: Conceptualization, methodology, writing – review & editing, supervision. BK: Writing – review & editing, funding acquisition

LS: Resources, data curation, writing – review & editing. RN: Conceptualization, writing – review & editing, supervision, funding acquisition. PM: Conceptualization, methodology, writing – review & editing, supervision, funding acquisition.

Handling co-editors: Keijo Mäkelä and Philippe Wagner

*Acta* thanks Johan Kärrholm and Stephan Maximilian Röhrl for help with peer review of this manuscript.
